# Supplementary material for: The association between cognitive ability and body mass index: A sibling-comparison analysis in four longitudinal studies
Source: PLoS Med. 2023 Apr 13;20(4):e1004207. doi: 10.1371/journal.pmed.1004207 (PMC10101525; doi:10.1371/journal.pmed.1004207)
Supplement: S1 Table — (DOCX) [file pmed.1004207.s003.docx]

|  | | Total | NLSY-79 Main | NLSY-79 Oversample | NLSY-79 CYA | NLSY-97 Main | NLSY-97 Oversample | WLS |
| --- | --- | --- | --- | --- | --- | --- | --- | --- |
| All Participants | Households | 29,515 (100%) | 4,012 (100%) | 3,478 (100%) | 4,941 (100%) | 5,184 (100%) | 1,630 (100%) | 10,270 (100%) |
|  | Individuals | 50,981 (100%) | 6,111 (100%) | 5,295 (100%) | 11,545 (100%) | 6,748 (100%) | 2,236 (100%) | 19,046 (100%) |
| Observed Sibling | Households | 14,257 (48.3%) | 1,279 (31.9%) | 857 (24.6%) | 3,068 (62.1%) | 1,249 (24.1%) | 424 (26%) | 7,380 (71.9%) |
|  | Individuals | 31,436 (61.7%) | 3,030 (49.6%) | 2,089 (39.5%) | 7,969 (69%) | 2,639 (39.1%) | 924 (41.3%) | 14,785 (77.6%) |
| Sibling Within +/- 5 Years | Households | 9,782 (68.6%) | 1,262 (98.7%) | 845 (98.6%) | 1,859 (60.6%) | 1,249 (100%) | 424 (100%) | 4,143 (56.1%) |
|  | Individuals | 21,013 (66.8%) | 2,936 (96.9%) | 1,998 (95.6%) | 4,219 (52.9%) | 2,639 (100%) | 924 (100%) | 8,297 (56.1%) |
| Aged 20+ by Final Follow-Up | Households | 9,726 (99.4%) | 1,262 (100%) | 845 (100%) | 1,803 (97%) | 1,249 (100%) | 424 (100%) | 4,143 (100%) |
|  | Individuals | 20,889 (99.4%) | 2,936 (100%) | 1,998 (100%) | 4,095 (97.1%) | 2,639 (100%) | 924 (100%) | 8,297 (100%) |
| Observed BMI @ Age 20+ | Households | 6,665 (68.5%; 68.5%) | 1,245 (98.7%; 98.7%) | 830 (98.2%; 98.2%) | 1,321 (73.3%; 73.3%) | 1,141 (91.4%; 91.4%) | 408 (96.2%; 96.2%) | 1,720 (41.5%; 41.5%) |
|  | Individuals | 14,560 (69.7%; 69.7%) | 2,888 (98.4%; 98.4%) | 1,954 (97.8%; 97.8%) | 2,978 (72.7%; 72.7%) | 2,409 (91.3%; 91.3%) | 887 (96%; 96%) | 3,444 (41.5%; 41.5%) |
| Observed Cognitive Ability | Households | 6,011 (90.2%; 61.8%) | 1,184 (95.1%; 93.8%) | 790 (95.2%; 93.5%) | 1,251 (94.7%; 69.4%) | 943 (82.6%; 75.5%) | 312 (76.5%; 73.6%) | 1,531 (89%; 37%) |
|  | Individuals | 13,146 (90.3%; 62.9%) | 2,737 (94.8%; 93.2%) | 1,859 (95.1%; 93%) | 2,819 (94.7%; 68.8%) | 1,990 (82.6%; 75.4%) | 675 (76.1%; 73.1%) | 3,066 (89%; 37%) |
| Observed Covariates | Households | 5,687 (94.6%; 58.5%) | 1,118 (94.4%; 88.6%) | 751 (95.1%; 88.9%) | 1,251 (100%; 69.4%) | 895 (94.9%; 71.7%) | 299 (95.8%; 70.5%) | 1,373 (89.7%; 33.1%) |
|  | Individuals | 12,427 (94.5%; 59.5%) | 2,569 (93.9%; 87.5%) | 1,760 (94.7%; 88.1%) | 2,819 (100%; 68.8%) | 1,885 (94.7%; 71.4%) | 645 (95.6%; 69.8%) | 2,749 (89.7%; 33.1%) |
| Observed SEP | Households | 5,668 (99.7%; 58.3%) | 1,114 (99.6%; 88.3%) | 750 (99.9%; 88.8%) | 1,251 (100%; 69.4%) | 889 (99.3%; 71.2%) | 291 (97.3%; 68.6%) | 1,373 (100%; 33.1%) |
|  | Individuals | 12,383 (99.6%; 59.3%) | 2,559 (99.6%; 87.2%) | 1,755 (99.7%; 87.8%) | 2,819 (100%; 68.8%) | 1,873 (99.4%; 71%) | 628 (97.4%; 68%) | 2,749 (100%; 33.1%) |
| Discordant Cognitive Ability (Final Sample) | Households | 5,602 (98.8%; 57.6%) | 1,113 (99.9%; 88.2%) | 750 (100%; 88.8%) | 1,246 (99.6%; 69.1%) | 889 (100%; 71.2%) | 291 (100%; 68.6%) | 1,313 (95.6%; 31.7%) |
|  | Individuals | 12,250 (98.9%; 58.6%) | 2,556 (99.9%; 87.1%) | 1,755 (100%; 87.8%) | 2,809 (99.6%; 68.6%) | 1,873 (100%; 71%) | 628 (100%; 68%) | 2,629 (95.6%; 31.7%) |
|  | Observations | 118,355 | 45,842 | 27,332 | 13,954 | 21,332 | 7,266 | 2,629 |
