# Supplementary material for: The association between cognitive ability and body mass index: A sibling-comparison analysis in four longitudinal studies
Source: PLoS Med. 2023 Apr 13;20(4):e1004207. doi: 10.1371/journal.pmed.1004207 (PMC10101525; doi:10.1371/journal.pmed.1004207)
Supplement: S2 Table — (DOCX) [file pmed.1004207.s004.docx]

|  | | | | Follow-Ups | | | | |
| --- | --- | --- | --- | --- | --- | --- | --- | --- |
| Cohort | Group, Cognitive Ability | N | Observations | Mean | SD | Min. | Max. | % Observed |
| NLSY-79 Main | All | 2,556 | 45,842 | 17.94 | 4.73 | 1 | 22 | 85.3% (22.1) |
|  | Below Average | 1,276 | 22,886 | 17.94 | 4.63 | 1 | 22 | 85.3% (21.7) |
|  | Above Average | 1,280 | 22,956 | 17.93 | 4.83 | 1 | 22 | 85.3% (22.6) |
| NLSY-79 Oversample | All | 1,755 | 27,332 | 15.57 | 6.33 | 1 | 22 | 74.1% (29.8) |
|  | Below Average | 897 | 14,041 | 15.65 | 6.32 | 1 | 22 | 74.3% (29.7) |
|  | Above Average | 858 | 13,291 | 15.49 | 6.33 | 1 | 22 | 74% (30) |
| NLSY-79 CYA | All | 2,809 | 13,954 | 4.97 | 1.9 | 1 | 10 | 75.3% (21.7) |
|  | Below Average | 1,396 | 6,894 | 4.94 | 1.9 | 1 | 10 | 75.4% (21.6) |
|  | Above Average | 1,413 | 7,060 | 5 | 1.9 | 1 | 10 | 75.1% (21.8) |
| NLSY-97 Main | All | 1,873 | 21,332 | 11.39 | 3.12 | 1 | 14 | 85.8% (22.6) |
|  | Below Average | 936 | 10,507 | 11.23 | 3.11 | 1 | 14 | 85.6% (22.9) |
|  | Above Average | 937 | 10,825 | 11.55 | 3.12 | 1 | 14 | 86.1% (22.4) |
| NLSY-97 Oversample | All | 628 | 7,266 | 11.57 | 2.86 | 1 | 14 | 86.7% (20.4) |
|  | Below Average | 319 | 3,668 | 11.5 | 2.76 | 1 | 14 | 86.4% (19.9) |
|  | Above Average | 309 | 3,598 | 11.64 | 2.96 | 1 | 14 | 87% (20.9) |
| WLS | All | 2,629 | 2,629 | 1 | 0 | 1 | 1 | 100% (0) |
|  | Below Average | 1,314 | 1,314 | 1 | 0 | 1 | 1 | 100% (0) |
|  | Above Average | 1,315 | 1,315 | 1 | 0 | 1 | 1 | 100% (0) |
