# Supplementary material for: The association between cognitive ability and body mass index: A sibling-comparison analysis in four longitudinal studies
Source: PLoS Med. 2023 Apr 13;20(4):e1004207. doi: 10.1371/journal.pmed.1004207 (PMC10101525; doi:10.1371/journal.pmed.1004207)
Supplement: S3 Table — (DOCX) [file pmed.1004207.s005.docx]

| Cohort | Variable | Group, Cognitive Ability | Mean | Total SD | Between SD | Within SD | Min. | Max. |
| --- | --- | --- | --- | --- | --- | --- | --- | --- |
| NLSY-79 Main | Age | All | 36.7 | 10.8 | 4.0 | 10.4 | 20 | 62 |
|  |  | Below Average | 36.7 | 10.8 | 4.0 | 10.4 | 20 | 62 |
|  |  | Above Average | 36.7 | 10.8 | 4.1 | 10.4 | 20 | 62 |
|  | BMI | All | 26.5 | 5.6 | 4.7 | 3.0 | 13.2 | 69.5 |
|  |  | Below Average | 26.6 | 5.7 | 4.9 | 3.1 | 13.4 | 68.7 |
|  |  | Above Average | 26.4 | 5.5 | 4.6 | 3.0 | 13.2 | 69.5 |
|  | Year | All | 1998.1 | 10.6 | 3.9 | 10.2 | 1981 | 2018 |
|  |  | Below Average | 1998.1 | 10.6 | 3.9 | 10.2 | 1981 | 2018 |
|  |  | Above Average | 1998.1 | 10.6 | 4.0 | 10.2 | 1981 | 2018 |
| NLSY-79 Oversample | Age | All | 36.1 | 10.9 | 5.7 | 10.2 | 20 | 61 |
|  |  | Below Average | 36.2 | 11.0 | 5.7 | 10.2 | 20 | 61 |
|  |  | Above Average | 36.0 | 10.9 | 5.7 | 10.1 | 20 | 61 |
|  | BMI | All | 27.4 | 6.1 | 5.1 | 3.3 | 13.2 | 68.7 |
|  |  | Below Average | 27.3 | 6.1 | 5.1 | 3.3 | 13.2 | 68.7 |
|  |  | Above Average | 27.5 | 6.1 | 5.2 | 3.3 | 14.8 | 68.7 |
|  | Year | All | 1997.5 | 10.7 | 5.5 | 10.0 | 1981 | 2018 |
|  |  | Below Average | 1997.5 | 10.8 | 5.5 | 10.1 | 1981 | 2018 |
|  |  | Above Average | 1997.5 | 10.7 | 5.5 | 10.0 | 1981 | 2018 |
| NLSY-79 CYA | Age | All | 26.4 | 4.9 | 2.5 | 4.4 | 20 | 46 |
|  |  | Below Average | 26.3 | 4.9 | 2.4 | 4.3 | 20 | 46 |
|  |  | Above Average | 26.4 | 4.9 | 2.5 | 4.4 | 20 | 46 |
|  | BMI | All | 27.2 | 6.3 | 5.6 | 2.6 | 13.6 | 66.4 |
|  |  | Below Average | 27.2 | 6.3 | 5.7 | 2.6 | 13.6 | 63 |
|  |  | Above Average | 27.2 | 6.2 | 5.6 | 2.7 | 14.4 | 66.4 |
|  | Year | All | 2010.3 | 5.2 | 3.6 | 4.2 | 1994 | 2018 |
|  |  | Below Average | 2010.3 | 5.2 | 3.6 | 4.1 | 1994 | 2018 |
|  |  | Above Average | 2010.2 | 5.2 | 3.6 | 4.2 | 1994 | 2018 |
| NLSY-97 Main | Age | All | 27.3 | 5.0 | 1.8 | 4.8 | 20 | 40 |
|  |  | Below Average | 27.2 | 5.0 | 1.8 | 4.8 | 20 | 40 |
|  |  | Above Average | 27.4 | 5.0 | 1.8 | 4.8 | 20 | 40 |
|  | BMI | All | 27.1 | 6.3 | 5.8 | 2.6 | 13.2 | 68.6 |
|  |  | Below Average | 27.2 | 6.3 | 5.7 | 2.6 | 13.2 | 68.6 |
|  |  | Above Average | 27.0 | 6.4 | 5.9 | 2.6 | 14.6 | 66.9 |
|  | Year | All | 2009.4 | 4.9 | 1.6 | 4.8 | 2002 | 2019 |
|  |  | Below Average | 2009.5 | 4.9 | 1.6 | 4.8 | 2002 | 2019 |
|  |  | Above Average | 2009.3 | 4.9 | 1.6 | 4.8 | 2002 | 2019 |
| NLSY-97 Oversample | Age | All | 27.5 | 5.0 | 1.6 | 4.8 | 20 | 40 |
|  |  | Below Average | 27.5 | 5.0 | 1.5 | 4.8 | 20 | 40 |
|  |  | Above Average | 27.5 | 5.0 | 1.6 | 4.9 | 20 | 40 |
|  | BMI | All | 28.7 | 6.6 | 5.9 | 2.9 | 14.1 | 64.8 |
|  |  | Below Average | 28.8 | 6.7 | 6.1 | 3.0 | 14.1 | 64.8 |
|  |  | Above Average | 28.5 | 6.5 | 5.7 | 2.9 | 14.1 | 61.3 |
|  | Year | All | 2009.4 | 4.9 | 1.4 | 4.8 | 2002 | 2019 |
|  |  | Below Average | 2009.5 | 4.9 | 1.3 | 4.8 | 2002 | 2019 |
|  |  | Above Average | 2009.4 | 4.9 | 1.5 | 4.8 | 2002 | 2019 |
| WLS | Age | All | 54.2 | 2.4 | 2.4 | 0.0 | 49 | 62.1 |
|  |  | Below Average | 54.2 | 2.4 | 2.4 | 0.0 | 49 | 61.2 |
|  |  | Above Average | 54.2 | 2.4 | 2.4 | 0.0 | 49.1 | 62.1 |
|  | BMI | All | 26.6 | 4.4 | 4.4 | 0.0 | 16.6 | 50.5 |
|  |  | Below Average | 26.7 | 4.5 | 4.5 | 0.0 | 16.6 | 50.5 |
|  |  | Above Average | 26.5 | 4.4 | 4.4 | 0.0 | 18 | 46.8 |
|  | Year | All | 1993.3 | 0.8 | 0.8 | 0.0 | 1992 | 1994 |
|  |  | Below Average | 1993.3 | 0.8 | 0.8 | 0.0 | 1992 | 1994 |
|  |  | Above Average | 1993.3 | 0.8 | 0.8 | 0.0 | 1992 | 1994 |
