# Supplementary material for: The association between cognitive ability and body mass index: A sibling-comparison analysis in four longitudinal studies
Source: PLoS Med. 2023 Apr 13;20(4):e1004207. doi: 10.1371/journal.pmed.1004207 (PMC10101525; doi:10.1371/journal.pmed.1004207)
Supplement: S4 Table — Analyses using first observation per individual. Between-family effect estimated using one randomly selected individual per household. Age, maternal age, birth order, sex, SEP, ethnic group, and cohort included as control variables in the first stage regressions with household fixed effects also included in within family models. Confidence intervals calculated using cluster-robust bootstrapping (percentile method, 500 replications). Columns headed excl. Refer to analyses excluding named cohort and indicate within-family associations. (DOCX) [file pmed.1004207.s006.docx]

| Quantile | Within Family | Between Family | excl. NLSY-79 Main | excl. NLSY-79 Oversample | excl. NLSY-79 CYA | excl. NLSY-97 Main | excl. NLSY-97 Oversample | excl. WLS |
| --- | --- | --- | --- | --- | --- | --- | --- | --- |
| 10th | 0.16 (-0.19, 0.44) | 0 (-0.35, 0.29) | 0.13 (-0.18, 0.51) | 0.06 (-0.23, 0.51) | 0.06 (-0.21, 0.43) | 0.16 (-0.18, 0.47) | 0.14 (-0.17, 0.45) | 0.18 (-0.23, 0.57) |
| 20th | 0.13 (-0.02, 0.47) | 0 (-0.24, 0.27) | 0.29 (-0.14, 0.56) | 0.2 (-0.06, 0.48) | 0.16 (-0.07, 0.51) | 0.12 (-0.12, 0.48) | 0.16 (-0.02, 0.47) | 0.13 (-0.21, 0.42) |
| 30th | 0.07 (-0.21, 0.39) | -0.23 (-0.47, 0.14) | -0.06 (-0.33, 0.27) | 0 (-0.24, 0.29) | 0.06 (-0.27, 0.46) | 0 (-0.29, 0.36) | 0.04 (-0.16, 0.4) | 0.14 (-0.02, 0.38) |
| 40th | -0.06 (-0.28, 0.15) | -0.22 (-0.56, 0.04) | 0 (-0.42, 0.33) | -0.02 (-0.35, 0.26) | -0.12 (-0.33, 0.21) | -0.07 (-0.34, 0.19) | -0.14 (-0.35, 0.18) | 0 (-0.25, 0.28) |
| 50th | -0.07 (-0.38, 0.33) | -0.29 (-0.66, 0.16) | -0.02 (-0.23, 0.17) | 0 (-0.37, 0.28) | -0.05 (-0.42, 0.27) | -0.05 (-0.39, 0.35) | -0.03 (-0.33, 0.28) | 0 (-0.38, 0.39) |
| 60th | 0 (-0.25, 0.16) | -0.28 (-0.79, 0.01) | 0 (-0.49, 0.36) | -0.02 (-0.61, 0.35) | -0.05 (-0.43, 0.34) | -0.03 (-0.29, 0.26) | -0.05 (-0.33, 0.25) | -0.02 (-0.28, 0.16) |
| 70th | -0.29 (-0.75, 0.07) | -0.56 (-1, -0.04) | -0.33 (-0.78, 0.31) | -0.1 (-0.78, 0) | -0.52 (-0.82, 0.27) | -0.32 (-0.81, 0.28) | -0.42 (-0.82, 0.18) | -0.18 (-0.7, 0.25) |
| 80th | -0.18 (-0.67, 0.31) | -0.52 (-1.15, 0.05) | -0.14 (-0.71, 0.44) | -0.29 (-0.83, 0.29) | -0.23 (-0.85, 0.39) | -0.25 (-0.77, 0.3) | -0.18 (-0.71, 0.38) | -0.06 (-0.67, 0.22) |
| 90th | -0.23 (-0.99, 0.47) | -0.5 (-1.44, 0.37) | -0.5 (-1.59, 0.34) | -0.49 (-1.2, 0.53) | -0.02 (-0.9, 0.77) | -0.53 (-1.25, 0.17) | -0.26 (-0.95, 0.47) | 0 (-1.21, 1.03) |
