# Supplementary figures and images for: The association between cognitive ability and body mass index: A sibling-comparison analysis in four longitudinal studies
Source: PLoS Med. 2023 Apr 13;20(4):e1004207. doi: 10.1371/journal.pmed.1004207 (PMC10101525; doi:10.1371/journal.pmed.1004207)

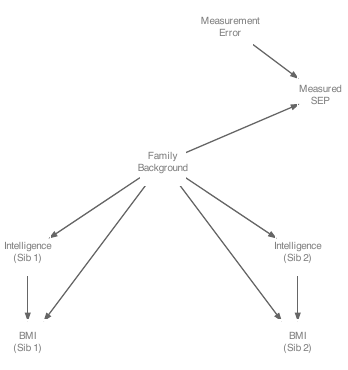

Supplement: S1 Fig — The association between (adolescent) cognitive ability and adult BMI may be confounded by family background (including dynastic genetic effects) and other factors shared between siblings. Existing studies typically attempt to control for family background with measured SEP, which, because of the few high-level variables that are used, can be thought of measuring family background with some measurement error. Thus, controlling for measured SEP does not full block confounding through family background. The sibling design instead accounts for family background by design. (TIFF) [file pmed.1004207.s007.tiff]

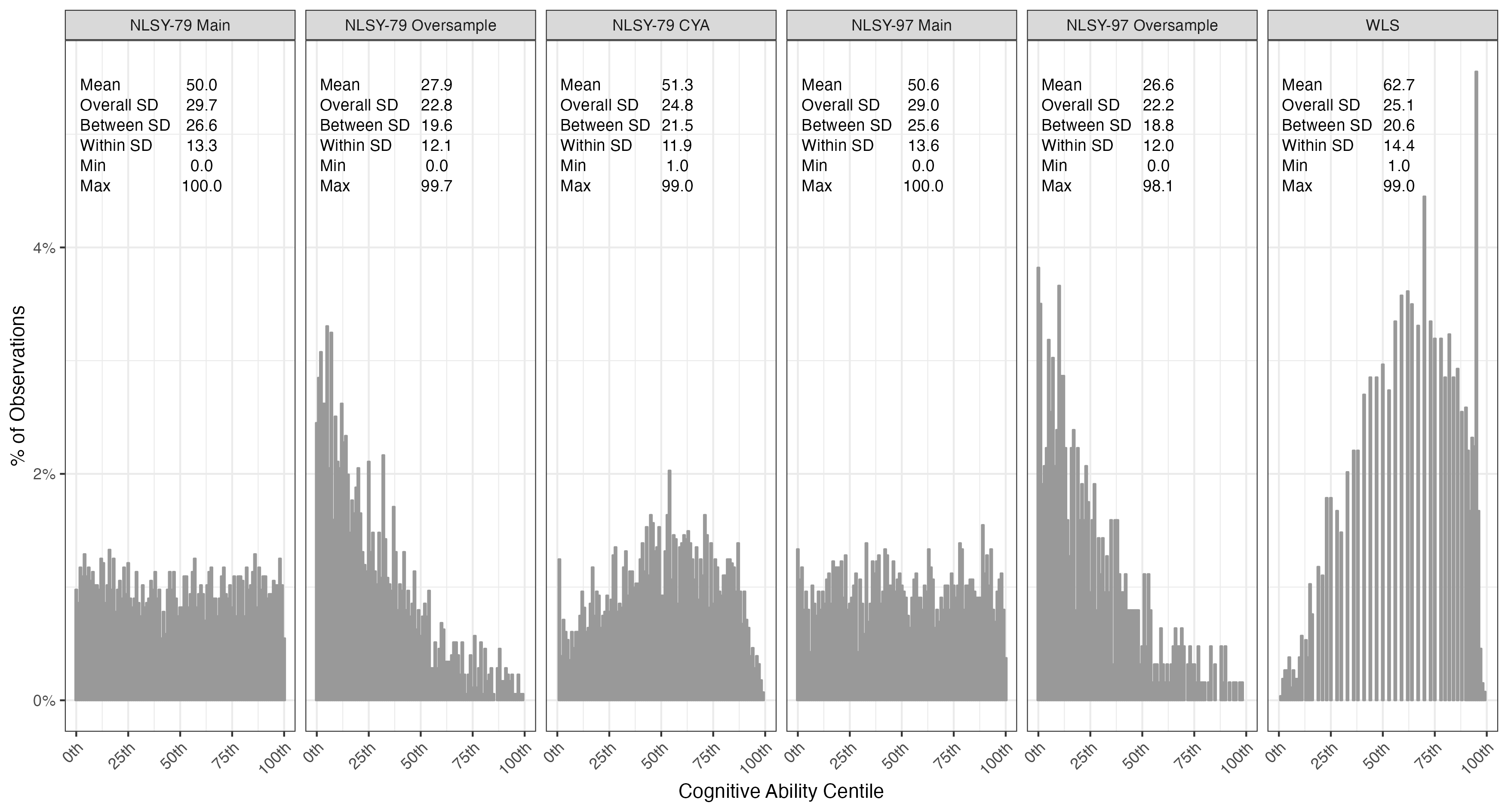

Supplement: S2 Fig — (TIFF) [file pmed.1004207.s008.tiff]

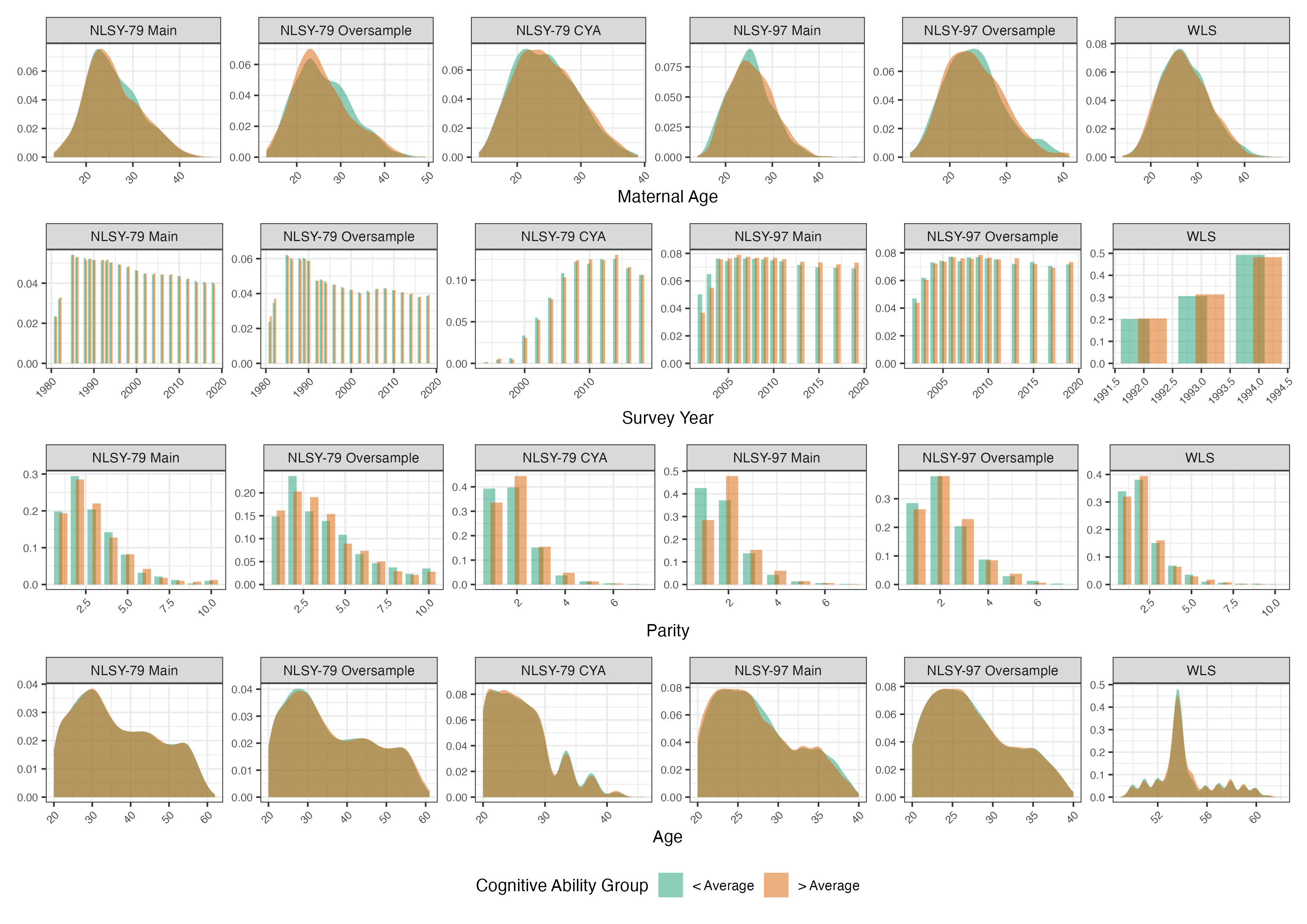

Supplement: S3 Fig — (TIFF) [file pmed.1004207.s009.tiff]

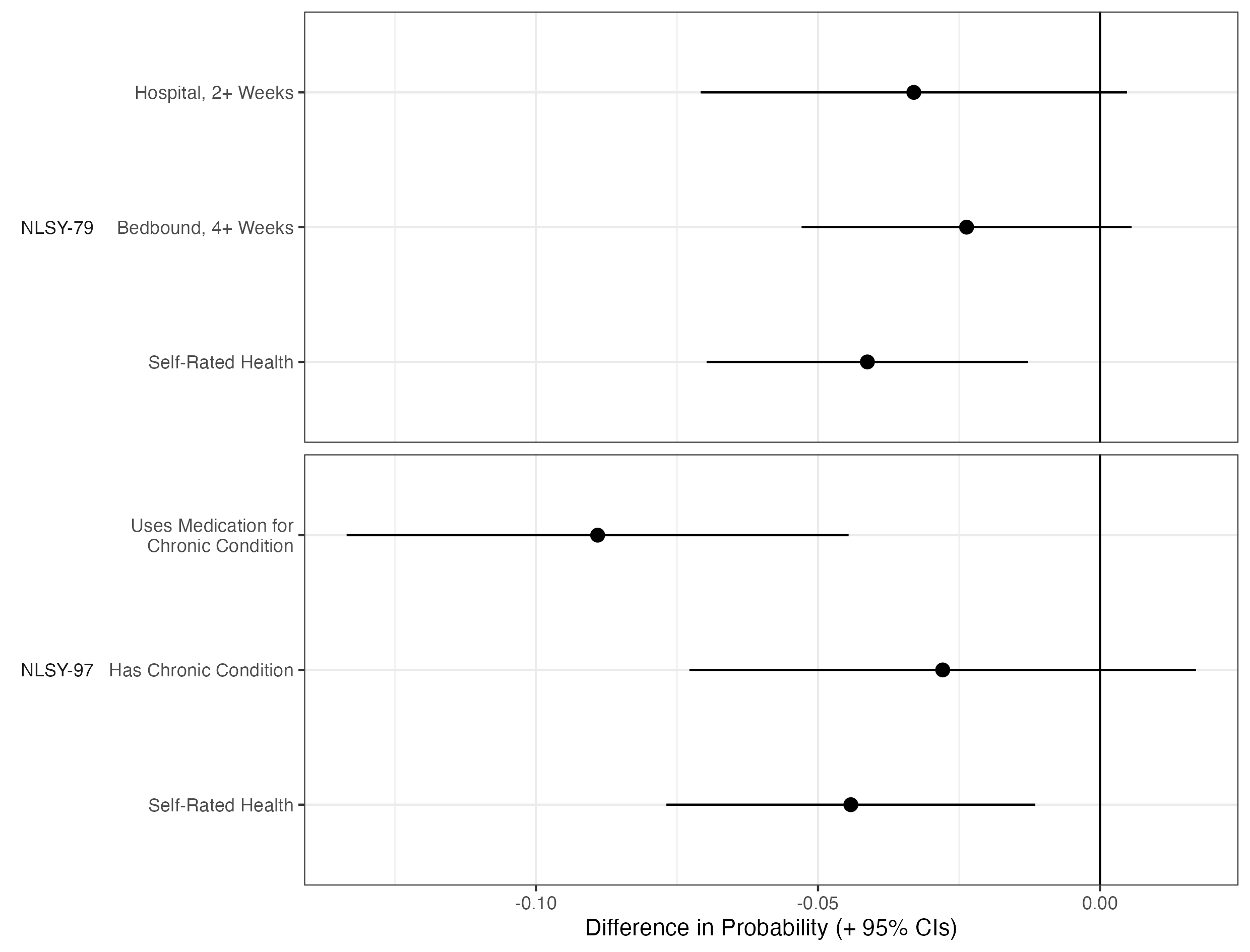

Supplement: S4 Fig — Estimates drawn from linear probability fixed effects models. Childhood health variables from NLSY-79 and NLSY-97. Self-rated health converted to binary variable for this analysis (poor or fair vs. good, very good, or excellent). Estimates show the difference in probability of each outcome as cognition centile increases from 25th to 75th centile. (TIFF) [file pmed.1004207.s010.tiff]

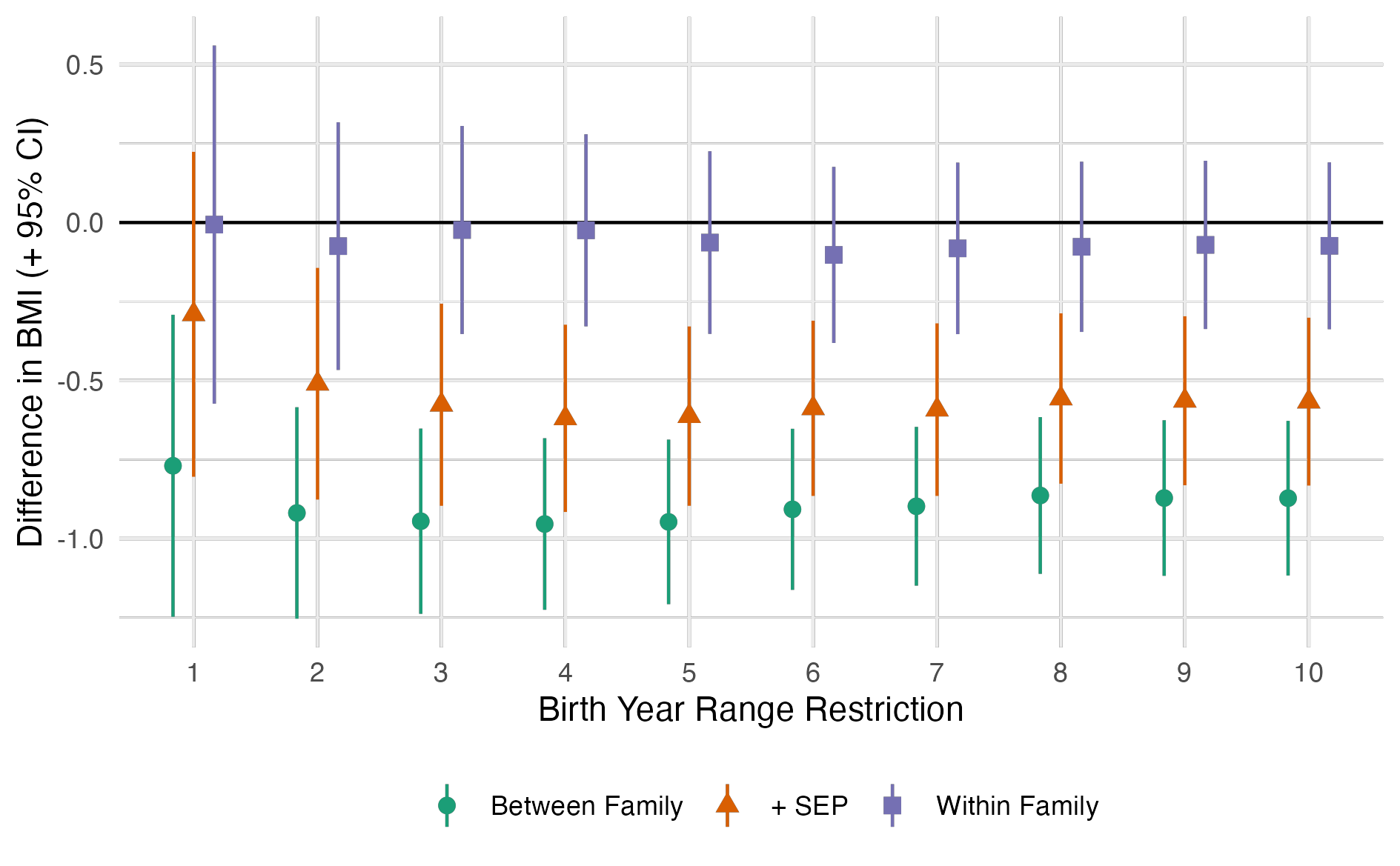

Supplement: S5 Fig — Derived from linear mixed effects models with random intercepts at the household and individual levels and age (two natural cubic splines), sex, cohort, birth order, ethnic group, and maternal age included as control variables. (TIFF) [file pmed.1004207.s011.tiff]

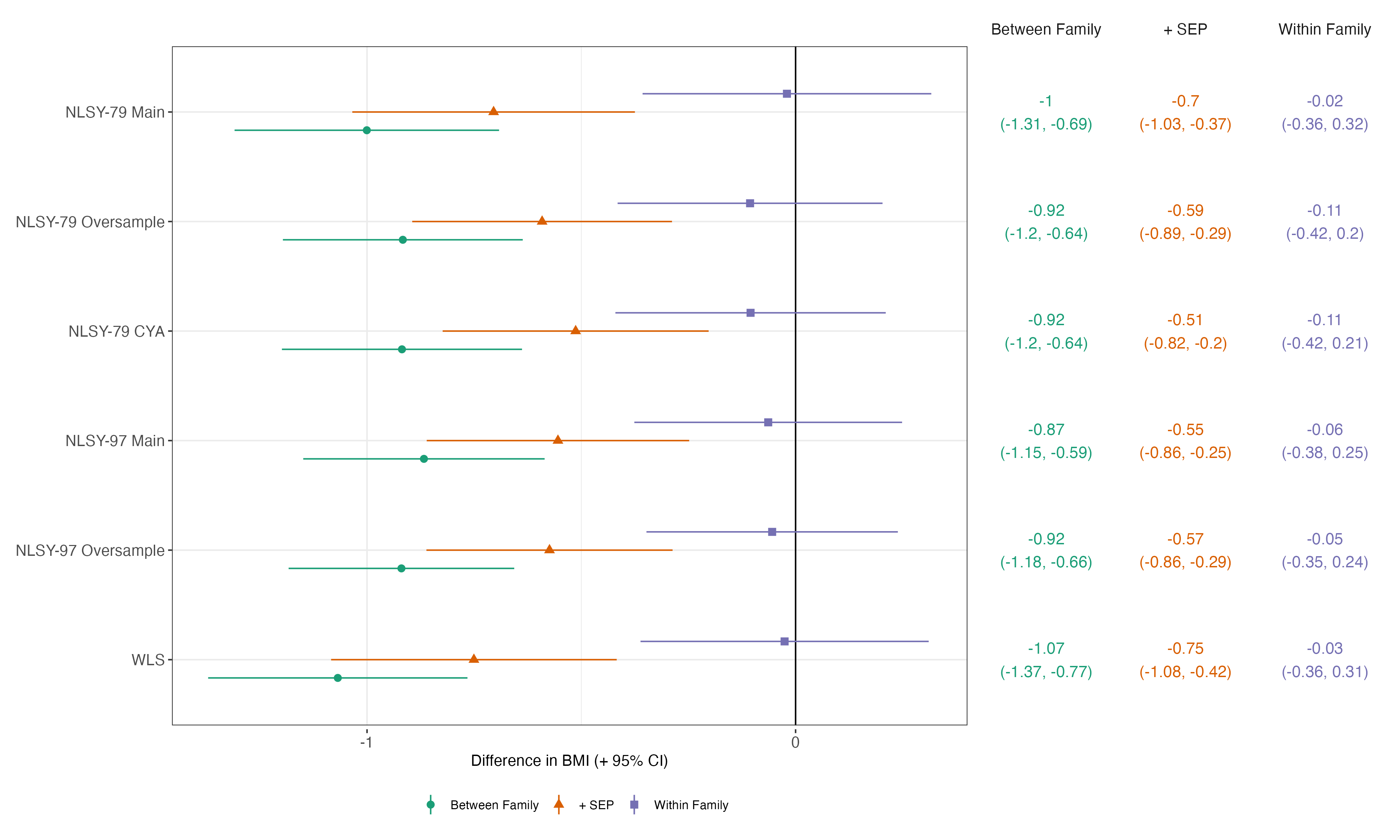

Supplement: S6 Fig — Models excluding the named cohort (y-axis). Estimates show predicted difference in BMI (kg/m2) comparing individuals at the 25th to 75th centiles of cognitive ability. Derived from linear mixed effects models with random intercepts at the household and individual levels and age (two natural cubic splines), sex, cohort, birth order, and maternal age included as control variables. (TIFF) [file pmed.1004207.s012.tiff]

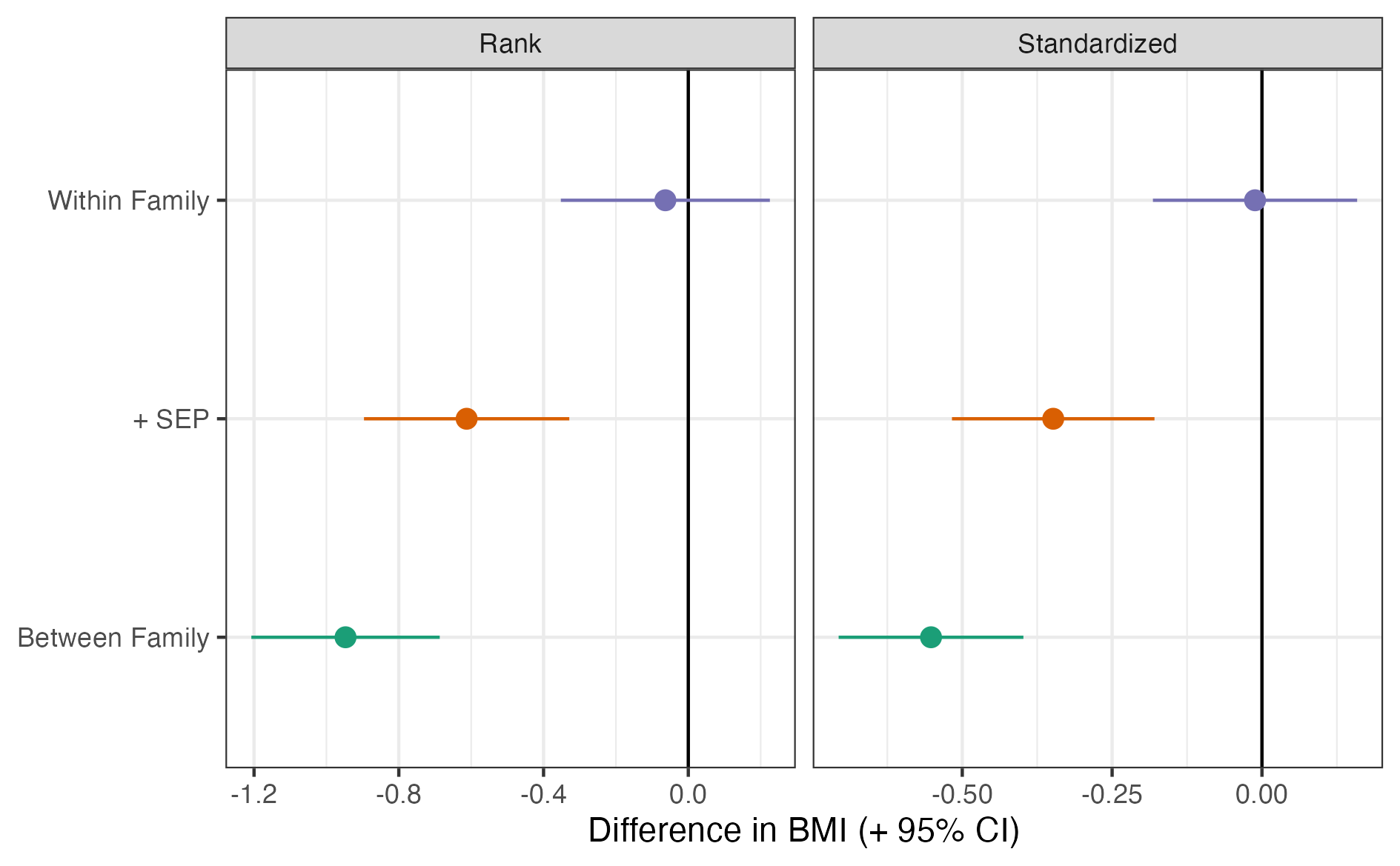

Supplement: S7 Fig — Derived from linear mixed effects models with random intercepts at the household and individual levels and age (two natural cubic splines), sex, cohort, birth order, ethnic group, and maternal age included as control variables. (TIFF) [file pmed.1004207.s013.tiff]

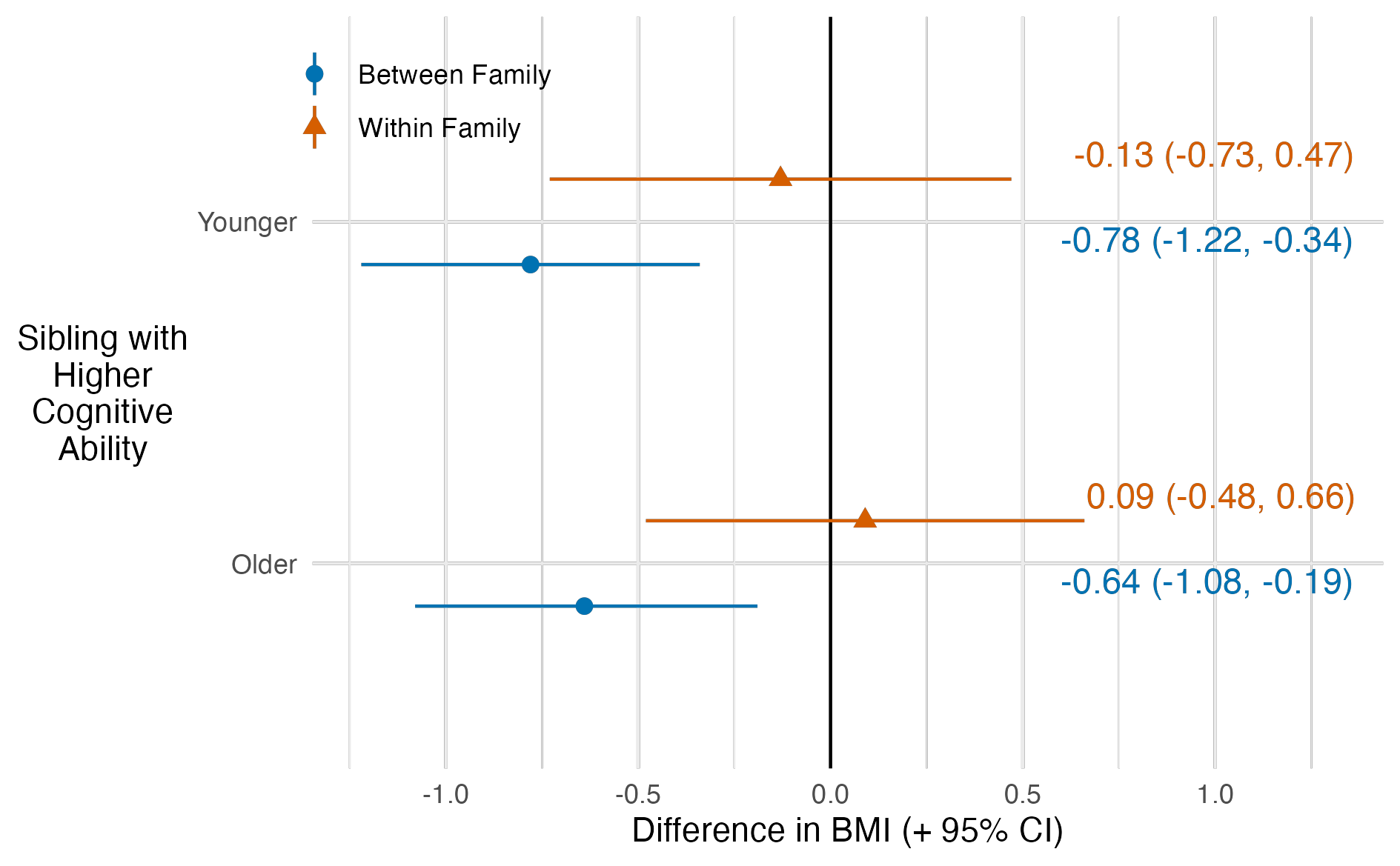

Supplement: S8 Fig — Derived from linear mixed effects models with random intercepts at the household and individual levels and age (two natural cubic splines), sex, cohort, birth order, ethnic group, and maternal age included as control variables. (TIFF) [file pmed.1004207.s014.tiff]

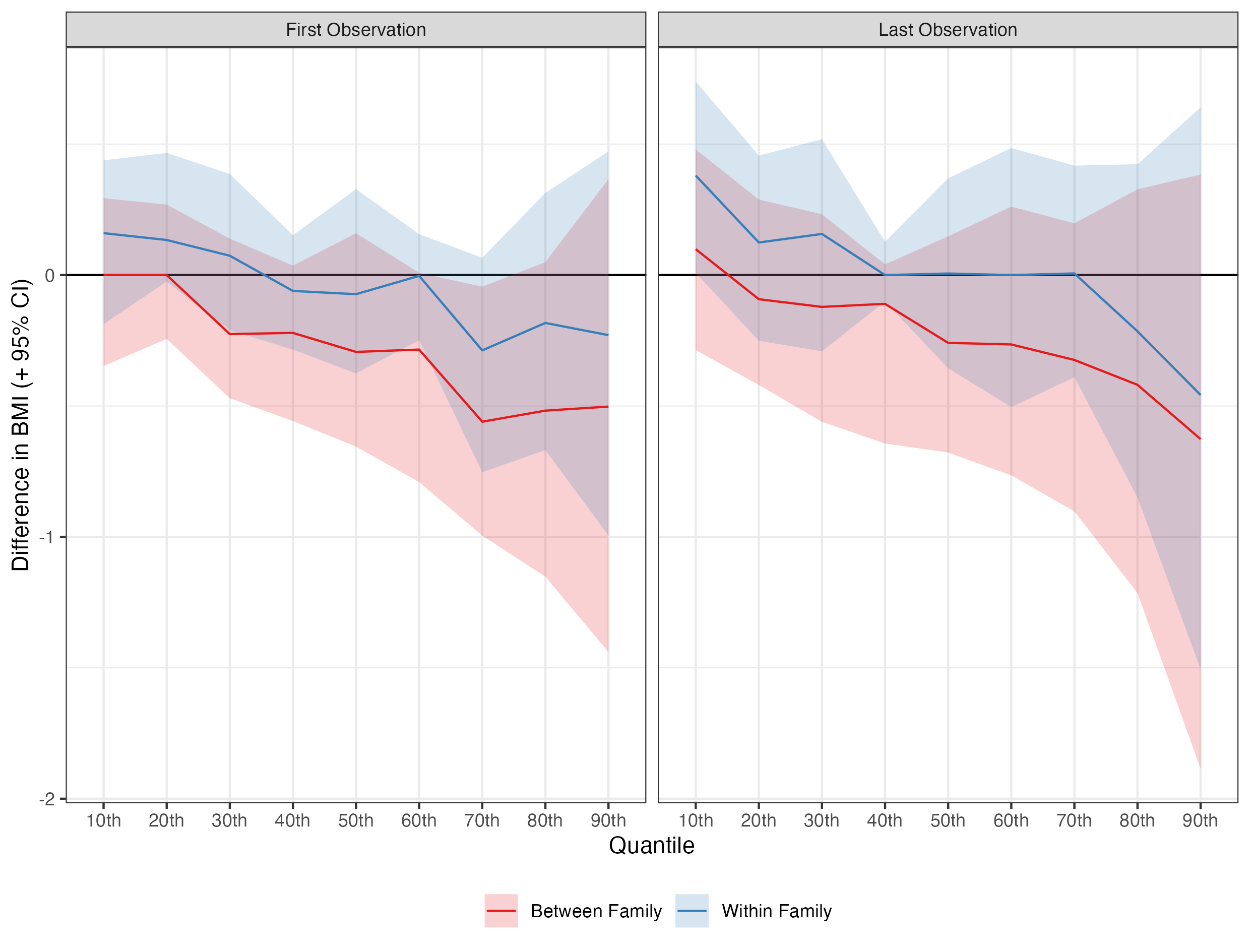

Supplement: S9 Fig — Derived from RQRs using the first (left panel) or last observation (right panel) per individual. Between-family effect estimated using one randomly selected individual per household. Age, maternal age, birth order, sex, SEP, ethnic group, and cohort included as control variables in the first stage regressions with household fixed effects also included in within-family models. Confidence intervals calculated using cluster-robust bootstrapping (percentile method, 500 replications). (TIFF) [file pmed.1004207.s015.tiff]
