# Supplementary material for: The association between cognitive ability and body mass index: A sibling-comparison analysis in four longitudinal studies
Source: PLoS Med. 2023 Apr 13;20(4):e1004207. doi: 10.1371/journal.pmed.1004207 (PMC10101525; doi:10.1371/journal.pmed.1004207)
Supplement: Attachment — Submitted filename: reviewer_response.docx [file pmed.1004207.s016.docx]

Dear Dr Davidson,

Thank you to you and the reviewers for reviewing our work. We are very pleased to read that you will accept our paper with revisions. We have made amendments to the manuscript based on your comments and believe it is improved as a result. Below we respond to each of the comments in turn.

First though, in the previous version of the paper, we used fewer control variables in the RQR models than the REWB models. We have now changed this for consistency. The results are not qualitatively changed (effect sizes in within family models continue to be weak).

With best wishes,

Liam Wright on behalf of all co-authors

### Editor Comments

| **Comment** | **Response** |
| --- | --- |
| Please structure your abstract using the PLOS Medicine headings (Background, Methods and Findings, Conclusions).  Abstract Methods and Findings:   - Please include the population and setting, length of follow up, and main outcome measures. - In the last sentence of the Abstract Methods and Findings section, please describe the main limitation(s) of the study's methodology. | The abstract has now been amended. |
| Please include continuous line numbering throughout your manuscript to facilitate further rounds of review. | These have been added to the manuscript |
| At this stage, we ask that you include a short, non-technical Author Summary of your research to make findings accessible to a wide audience that includes both scientists and non-scientists. The Author Summary should immediately follow the Abstract in your revised manuscript. This text is subject to editorial change and should be distinct from the scientific abstract. Please see our author guidelines for more information:  https://journals.plos.org/plosmedicine/s/revising-your-manuscript#loc-author-summary | This has now been added to Page 3 of the manuscript. |
| Please ensure that you cite items in the Supporting Information as described here:  https://journals.plos.org/plosmedicine/s/supporting-information | We now have separate files for the individual Supporting Information items. |
| Please define "lost to follow-up" as used in this study. Other reasons for exclusion should be defined. | In the Statistical Analysis subsection of the Method section, we now explain that participants were deemed lost to follow-up as soon as they provided no further BMI data. |
| Please define the length of follow up (eg, in mean, SD, and range) in the main text. | We have now added descriptive statistics for number of BMI measurements per individual to the Results section. We choose BMI measurement occasions, rather than time in the study, given differences in the age participants enter and exit the study across the cohorts used. |
| Please ensure the Y-axis scales match between Figures 2 and 3 to facilitate comparison. | Figures 2 and 3 are from two different models and are not directly comparable. Figure 2 shows the association between cognitive ability and *mean* BMI by age. Figure 3 shows the association between cognitive ability and the distribution of BMI. Given that they are not comparable, we have left the scales as is to make the individual figures as clear as possible. |
| Please enlarge the text in Figure 1 as it is currently too small to read. | Figure 1 has now been amended. |
| Please remove the ‘Declaration of interest’, ‘Funding’, ‘Author contributions’, and ‘Data Availability’ Sections from the end of the main text. All of this information is captured as metadata (to be published alongside the article in the event of acceptance) via the submission form questions. | These sections have now been removed |
| Please use ‘et al.’ in the references after listing the first six authors (this also applies to the Supporting Information). See https://journals.plos.org/plosmedicine/s/submission-guidelines#loc-references for further guidelines. | We now use PLOS Medicine citation style for our references. |
| Please ensure that the study is reported according to the STROBE guideline, and include the completed STROBE checklist as Supporting Information. Please add the following statement, or similar, to the Methods: "This study is reported as per the Strengthening the Reporting of Observational Studies in Epidemiology (STROBE) guideline (S1 Checklist)."  The STROBE guideline can be found here: http://www.equator-network.org/reporting-guidelines/strobe/  When completing the checklist, please use section and paragraph numbers, rather than page numbers. | We have now completed the STROBE Checklist (S1 Checklist). We noticed that we did not discuss generalizability in the manuscript previously, so have now added a sentence to the Strengths and Limitations section of the Discussion. Note, we have also extended the discussion of bias due to measurement error following discussion among the co-authors. |
| Did your study have a prospective protocol or analysis plan? Please state this (either way) early in the Methods section.  a) If a prospective analysis plan (from your funding proposal, IRB or other ethics committee submission, study protocol, or other planning document written before analyzing the data) was used in designing the study, please include the relevant prospectively written document with your revised manuscript as a Supporting Information file to be published alongside your study, and cite it in the Methods section. A legend for this file should be included at the end of your manuscript.  b) If no such document exists, please make sure that the Methods section transparently describes when analyses were planned, and when/why any data-driven changes to analyses took place.  ) In either case, changes in the analysis-- including those made in response to peer review comments-- should be identified as such in the Methods section of the paper, with rationale. | We confirm that a prospective protocol was not used in this secondary data analysis study. We have added details as instructed regarding the analysis planning (see revised Methods, Statistical analyses section). |

### Reviewer 1 Comments

| **Comment** | **Response** |
| --- | --- |
| The paper by Wright, Davies, and Bann considers the association between adolescent cognitive ability, and later adult BMI. They demonstrate that these associations are visible only in between-family analyses, and not within-family, suggesting that these associations are probably due to shared confounders within families. This review considers the statistical aspects of the paper.  The authors use a mixed effects model of continuous BMI, and a quantile regression method, suitable for the family data structure. They use multiple cohorts, and consider the sensitivity of the results to each cohort used. My comments are relatively minor. | Thank you for taking the time to review our work. |
| In the REWB models, the association is reported as the difference in adult BMI between the extremes of the cognitive ability distribution. This is an unnecessarily large difference - for me it would make more sense to report the difference between the 75th and 25th percentiles, i.e. the middle of the upper and lower halves of the distribution. | We have now presented results comparing 75^th^ vs 25^th^ centiles of cognitive ability. |
| The abstract uses "points" when "kg/m2" would be more accurate. | We have now changed this. |
| In the statistical methods section, age is reported as one of the adjustment variables. I assume this is age as an adult, when BMI is measured? This could be clearer. | That is correct. We’ve now updated the text in the methods section to make this clearer. |
| Also, maternal age and birth order are included in the models. Are these correlated? Within families, these must be quite strongly linked. Is that OK for these models? | Maternal age and birth order are highly correlated within families in the models. Given issues of multicollinearity, we reran the main REWB and RQR models including maternal age and birth order separately. Results were almost identical regardless of covariates included. |
| I disagree slightly with the interpretation of the quantile regression results. In the between-family analysis, it looks to me as though there is no (or very little) evidence of an association between adolescent cognitive ability and the location of the 10th percentile of the adult BMI distribution, whereas there does seem to be an association with higher percentiles of BMI. I have seen this before. The lower end of the BMI distribution is fairly fixed, but the risk factor (lower adolescent cognitive ability) is associated with a "stretching" of the BMI distribution in adulthood. Not required for this paper, but I wonder if this can be seen in the variance of adult BMI in relation to adolescent cognitive ability? | The RQR results are slightly changed as we have now added further control variables to be consistent across the REWB and RQR models (see above).  We have changed the discussion of the RQR results to reflect your observation that effect sizes are stronger at higher deciles of BMI.  Like you, we [have](https://doi.org/10.1371/journal.pgen.1010233) [observed](https://doi.org/10.7554/eLife.72357) that risk factors for BMI have stronger associations at the top end of the distribution (i.e., increasing the risk of obesity but not reducing the likelihood of underweight).  To check whether cognitive ability is related to the variance of BMI (between families), we ran a GAMLSS model with BMI modelled to follow a normal distribution and mean and standard deviation allowed to differ according to cognitive ability and the other control variables used in the RQR and REWB models presented in the paper. Higher cognitive ability was indeed related to lower variance – moving from 25^th^ to 75^th^ centiles of cognitive ability was related to approximately 6% lower SD of BMI. |

### Reviewer 2 Comments

| **Comment** | **Response** |
| --- | --- |
| This is a very nicely written sibling analysis of cognitive ability and BMI. The association between higher cognitive ability and lower BMI does not hold when comparing siblings, which suggest family confounding. The data comes from 4 cohort studies, which provides sufficient evidence base. The methods and results are clearly described, and the results are interpreted appropriately. I didn't find anything to improve in the manuscript. | Thank you for your kind comments. |

### Reviewer 3 Comments

| **Comment** | **Response** |
| --- | --- |
| This important paper reports large-scale within-family analysis of associations between cognitive test performance in adolescence and body mass index (BMI) in adulthood using data from about 5000 families in the United States. The key findings is that the association between cognitive performance and BMI is substantially attenuated when comparisons are made between siblings within a family. This attenuation is nearly complete (effect sizes are quite close to zero in the within-family analysis) and much greater than in models that use statistical methods to adjust for socioeconomic differences between families.  Cognitive test performance and body mass index are negatively correlated. In neurology, this association has been explored as suggesting potential brain-damaging effects of obesity. In life-course epidemiology, the same association has been explored as suggesting effects of cognitive abilities on obesity risk. Overall, there is more evidence to support the latter of these interpretations; in longitudinal studies, differences in cognitive performance tend to precede differences in body mass index. However, the mechanism through which an association between cognitive performance and obesity comes about is unclear. In this article the authors show convincing evidence that differences between the families of high- and low- cognitive performance individuals account for the gradient.  The sibling comparison design used in this study is a powerful method to separate effects that arise from an individual's own cognitive characteristics from those that arise from family-level factors correlated with those characteristics, including parents' education and income, health behaviors, values, etc. | Thank you for your comments. |
| My one comment about the paper regards how the authors interpret their data. To my mind, the authors lean too heavily on family socioeconomic differences as the confounder driving cognitive performance associations with BMI. Covariate adjustment for measures of family SES do not account for large fractions of the association between cognitive performance and BMI. I fully agree with the authors that measurement error may lead to incomplete control for SES in the covariate-adjusted between-family models. But the much larger attenuation seen in the within-family analyses suggests that there may be more to the family-level confounding of cognitive performance-BMI associations than socioeconomic status. It would enhance the article to include some discussion of what these other family level factors might be. | We have now expanded the discussion to note other confounders shared between siblings: “Besides SEP, other possible confounders shared between siblings include parenting practices and early neighbourhood (social and built) environments, which may influence the formation of diet and exercise habits, and maternal obesity which may influence development in utero [63] and, subsequently, offspring BMI into adulthood (at least for extreme maternal obesity) [64]. Further, given that siblings share on average 50% of their genes with other siblings and 50% with each parent, associations could therefore be confounded by shared genetic factors which influence cognition and BMI [65].” |
| Importantly, the authors note that public health messaging around diet and exercise seem to have limited impact on adults' obesity risk. One interpretation of the data reported in this paper is that such messaging may nevertheless impact the way parents raise their children. | We have added to the discussion to highlight this possibility. “Though our results suggest a limited causal role for cognitive ability on one’s own BMI, it’s possible that parental cognitive ability still has importance [44] through influencing family SEP and parenting practices (public health interventions could have indirect effects through parental practices, too).” |

### Reviewer 4 Comments

| **Comment** | **Response** |
| --- | --- |
| This is an interesting study testing between- and within-family associations between adolescent / young adult cognitive ability and BMI measured between ages 20 and 60 years. The study takes advantage of four different cohorts resulting in 2,250 siblings from 5,602 households. The study found in a between-family comparison that higher cognitive ability was associated with lower BMI, and family socioeconomic position attenuated this association slightly. However, in a within-sibling comparison cognitive ability and BMI were not associated, suggesting that the associations are explained by unmeasured familial (shared environmental or genetic) factors.  The authors have used recently developed state of the art statistical analyses to address the study questions. While this is a well-written manuscript, which conveys an important message, there are, however, a few concerns. | Thank you for your comments. |
| Most important one relates to self-reported height and weight. While the authors have discussed this briefly as a study limitation, they have not addressed - in their analyses or discussion - that, according to some studies self-reports of weight and height carry a gender/sex bias, women more often underreport their weight and overreport their hight compared with men.  This may to some extent bias the sibling-comparisons - in sibling pairs discordant for sex. While the models have covaried for sex, I would suggest carrying out stratified sub-group analyses with sex-concordant and sex-discordant sibling pairs. This at least would reduce some of the reporting bias. | We repeated the main REWB models for sex-concordant and sex-discordant siblings. The results are similar in each case. We have now referred to this analysis and its results in the Statistical Analysis section of the manuscript. |
| Table 1 is very difficult to comprehend, what do below average and above average refer to? The numbers M(SD), N(%) do not make much sense. This needs further clarification. Alternatively, you may want to consider just showing characteristics of your cohorts and total study population without reference to below and above average, would make more sense to me, and would allow more straightforward comparison to other studies. | In Table 1, below average refers to siblings whose cognitive ability is lower than the average cognitive ability for *their* household – in two sibling households, it is the sibling with the lower cognitive ability score. The purpose of Table 1 is to show that the below and above average groups are broadly balanced with respect to covariates – an assumption of the sibling comparison model is that there is no within family confounding. We have now included further explanation of the table in the table caption. |
| Please update your literature review. For instance, your literature on statistics of obesity and overweight prevalence and their disease burden is outdated (e.g. 10.1371/journal.pmed.1003198). | We have now included more recent references in our literature review. In some instances, we have cited older work to reflect the particular statistics we are discussing (e.g., the literature on changes in median BMI). |
| Please attenuate your conclusion about family SEP, as it is on the same pathway than cognitive ability; hence, an alternative interpretation of the attenuated associations between cognitive ability and BMI in analyses including SEP is that SEP is a mediator? | Family SEP was measured using socioeconomic data (education, income, etc.) from participants’ parents when the participants were still adolescents. Adolescents are unlikely to have a significant effect on parents’ socioeconomic circumstances, so family SEP is unlikely to mediate the effect that cognitive ability has on a person’s own BMI when they are adults.  Family SEP could mediate the influence parental cognitive ability may have on offspring BMI, however. We now note this in the discussion, where we write “Though our results suggest a limited role for one’s own cognitive ability on BMI, parental cognitive ability could still have importance [44] by influencing family SEP and parenting practices regarding health behaviour (public health messaging could have effects through parental practices, too).” |
