# Supplementary material for: The association between cognitive ability and body mass index: A sibling-comparison analysis in four longitudinal studies
Source: PLoS Med. 2023 Apr 13;20(4):e1004207. doi: 10.1371/journal.pmed.1004207 (PMC10101525; doi:10.1371/journal.pmed.1004207)
Supplement: Attachment — Submitted filename: reviewer_response.docx [file pmed.1004207.s017.docx]

Dear Dr Davidson,

Thank you to you and the reviewers for reviewing our work. We are delighted that you will accept our paper. Below are our responses to the remaining comments.

With best wishes,

Liam Wright on behalf of all co-authors

### Editor Comments

| **Comment** | **Response** |
| --- | --- |
| Please include headline numbers in your Author Summary (including sample size and key findings). | We have now added the sample size and the result from the main (within-sibling) regression analysis to the Author Summary |
| Please ensure your STROBE checklist uses section headings and paragraph numbers. Any other identifier (e.g., page or line number) is likely to change during the publication process. | We have changed the column header from “Page” to “Section” in our completed STROBE checklist (S1 Checklist) |
| References 3, 12, 19, 42, and 46 contain details of the academic editors which can be removed. | These have now been amended |
| Please include the date accessed for internet sources in your references. | Accessed dates have been added to the relevant references. |
| References 49 and 57 are preprints – please confirm these studies are still preprints and, if so, please add [preprint] per our formatting guidelines: https://journals.plos.org/plosmedicine/s/submission-guidelines#loc-references | We have updated these references. ([49] is still a preprint; [57] has now been published.) |
| Please check that the references in your Supporting Information are presented according to the guidance at the link above. | We have updated references in the Supporting Information to the PLOS Medicine style. |
| To help us extend the reach of your research, please provide any Twitter handle(s) that would be appropriate to tag, including your own, your coauthors’, your institution, funder, or lab. Please respond to this email with any handles you wish to be included when we tweet this paper. | @davidbann; @nm_davies |

### Reviewer 1 Comments

| **Comment** | **Response** |
| --- | --- |
| I thank the authors for their consideration of my original points. I am happy with their responses, and have no further comments to make. | Thank you for taking the time to review our work. |

### Reviewer 4 Comments

| **Comment** | **Response** |
| --- | --- |
| The authors have done a good job in answering the concerns that this reviewer had. However, regarding Table 1, which has been clarified, I continue having difficulties in understanding the rows related to households, and why the Ns are constant across total, below average and above average. Perhaps you could add a footnote, which would clarify this detail. | Thank you for your comments. We have now added further explanation to the table caption. |
